# Simulation and Prediction of Ion Transport in the Reclamation of Sodic Soils with Gypsum Based on the Support Vector Machine 

**DOI:** 10.1155/2014/805342

**Published:** 2014-03-16

**Authors:** Jinman Wang, Zhongke Bai, Peiling Yang

**Affiliations:** ^1^College of Land Science and Technology, China University of Geosciences, 29 Xueyuanlu, Handian District, Beijing 100083, China; ^2^Key Laboratory of Land Consolidation and Rehabilitation, Ministry of Land and Resources, 37 Guanying Park West, Xicheng, Beijing 100035, China; ^3^College of Hydraulic and Civil Engineering, China Agricultural University, 17 Qinghua Donglu, Handian District, Beijing 100083, China

## Abstract

The effect of gypsum on the physical and chemical characteristics of sodic soils is nonlinear and controlled by multiple factors. The support vector machine (SVM) is able to solve practical problems such as small samples, nonlinearity, high dimensions, and local minima points. This paper reports the use of the SVM regression method to predict changes in the chemical properties of sodic soils under different gypsum application rates in a soil column experiment and to evaluate the effect of gypsum reclamation on sodic soils. The research results show that (1) the SVM soil solute transport model using the Matlab toolbox represents the change in Ca^2+^ and Na^+^ in the soil solution and leachate well, with a high prediction accuracy. (2) Using the SVM model to predict the spatial and temporal variations in the soil solute content is feasible and does not require a specific mathematical model. The SVM model can take full advantage of the distribution characteristics of the training sample. (3) The workload of the soil solute transport prediction model based on the SVM is greatly reduced by not having to determine the hydrodynamic dispersion coefficient and retardation coefficient, and the model is thus highly practical.

## 1. Introduction

The support vector machine (SVM) method was developed based on the Vapnik statistical learning theory and in particular the statistical Vapnik-Chervonenkis Dimension theory and the Structural Risk Minimization Inductive Principle [1]. The basic principle of SVM is to solve the classification problem. Its guiding ideology is to map nonlinear separable sample data to a higher dimensional linear separable space through a kernel function and then to solve the partition hyperplane using an optimization method to determine the parameters of the decision-making function and minimize its structural risk [2]. Theory and practice have proved that the SVM can solve the practical problems of a small sample, nonlinearity, high dimensionality, and local minima points and has good generalizability [3]. The SVM has become a hot topic in machine learning research and has been successfully applied in pattern recognition, data mining, regression, and approximation [4–6]. In the field of agriculture, the problem of reclaiming sodic soils has attracted much attention. Many studies have proved that the effect of the desulfurization byproducts used in the reclamation process on the physical and chemical properties of soil is nonlinear and multifactorial [6–11]. Šimůnek et al. constructed a multicomponent solute transport model of sodic soil reclamation and predicted the solute transport by using the UNSATCHEM and HYDRUS models [12–14]. However, the models use the major ion chemistry module, which have a relatively complex code and require a large number of input parameters that are not always readily available [15–17]. The SVM regression method is applied here to overcome these issues and determine the variation in the chemical properties of sodic soils with reclaimed time and different application levels of a flue gas desulfurization byproduct (or gypsum) and to evaluate the effect on sodic soils of reclamation with gypsum.

## 2. Materials and Methods

### 2.1. Physical and Chemical Properties of Soils and CaSO_4_

The soil was sampled from the Changsheng Experimental Station of the Bayannur League Institute of Water Resources in the northwest of China. The soil had typical sodic soil characteristics, that is, a high pH and exchangeable sodium percentage and low hydraulic conductivity. The soil texture was clay and its physical and chemical properties are listed in Table 1. The soil was air-dried, crushed, and passed through a 2 mm sieve before the experiments. The pure CaSO_4_ (molecular weight 136) was purchased from the Tianjin Wendaxigui Chemical Reagent Factory.

### 2.2. Ca^2+^ Penetration Experiment

**Figure 1:**
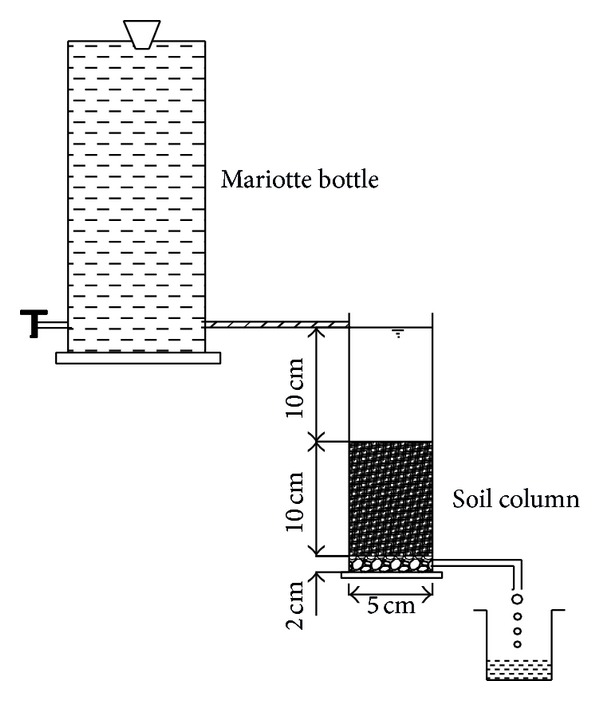
Experimental device of Ca^2+^ penetration.

The experimental device (Figure 1) was a 25-cm-high Plexiglass column with a 5 cm inside diameter (the soil height was 10 cm), and a Mariotte bottle was used to supply the water. The experiment consisted of four treatments (Table 2) with different CaSO_4_ rates (0.5, 1, 1.5, and 2 g·L^−1^). The pure CaSO_4_ was selected as the reagent and distilled water was used as the solvent to configure the solution.

The experimental procedure was as follows. The tested soil samples were poured homogeneously into the Plexiglass column at a dry soil bulk density of 1.45 g·cm^−3^ to a depth of 10 cm. The soil columns were saturated with distilled water from the bottom up at a saturation rate of 2 cm per hour. Once the soils were saturated, the surface water was quickly drained with a vacuum pump and the Ca^2+^ solution that had been configured in the Mariotte bottle was immediately supplied. The Ca^2+^ and Na^+^ concentrations of the leachate were measured once every 24 hours. The Ca^2+^ and Na^+^ concentrations in the soil solution and soil colloid were measured after the penetration experiment.

### 2.3. Analytical Methods and Statistical Analyses

The soil samples were air-dried and passed through a 1 mm sieve. The EC, pH, soluble anions, and soluble cations were measured using 1 : 5 water extracts. The soluble cations were measured using an atomic absorption spectrophotometer (AAS-3620); soluble anions were determined by anion chromatography (ICS-900); soil pH was determined with the glass electrode method. The salt content was measured using a 1 cm conductivity cell, dip-type probe. Exchangeable cations were determined in a 1 M ammonium acetate (pH = 7) extract. Following this extraction and washing with 96% alcohol, the cation exchange capacity was determined by the removal of ammonium ions by distillation. Na^+^ and K^+^ were determined by flame emission spectroscopy (FP6400) in the extract, and Ca^2+^ and Mg^2+^ were determined by atomic absorption spectrophotometer (AAS-3620). Particle size distribution was determined with the hydrometer method. The concentrations of Na^+^ and Ca^2+^ in the leachate were measured using an atomic absorption spectrophotometer (AAS-3620).

### 2.4. SVM Theory

The SVM method was initially introduced to solve classification problems but can be extended to deal with regression problems [6, 11].

A linear and separable sample set (*x*_*i*_ ∈ *R* ^*n*^, *y*_*i*_ ∈ {1, −1}, *i* = 1,…, *l*) is defined, and the hyperplane (w* · *x*) + *b* = 0 is constructed and divided. The two types of sample points must be correctly divided to achieve *y*_*i*_((*w* · *x*_*i*_) + *b*) ≥ 1,  *i* = 1,…, *l*, but the largest spacing of classifications needs to be achieved, where 2/||*w*|| is the maximum or (1/2)||*w*||^2^ is minimum. w* and b* give the optimal solution to the following optimization problem:

(1)  min⁡w,b ⁡12||w||2,s.t. yi((w·xi)+b)≥1, i=1,…,l.

This is the decision-making function *f*(*x*) = sgn⁡((w* · *x*) + b*).

Equation (1) can be transformed into the dual problem based on duality theory [6]:

(2) $$\begin{array}{c} \underset{\alpha }{\min}\;\frac{1}{2}\sum_{i=1}^{l}\sum_{j=1}^{l}y_{i}y_{j}\alpha_{i}\alpha_{j}\left(x_{i}\cdot x_{j}\right)-\sum_{j=1}^{l}\alpha_{j},\\ \;\text{s}.\text{t}.\sum_{i=1}^{l}y_{i}\alpha_{i}=0,\;\alpha_{i}\geq 0,\;i=1,\ldots ,l, \end{array}$$

where *α*_*i*_ is a Lagrange multiplier. The following relationship exists among the optimal solutions α* in (2) and w* and b* in (1):

(3) $$\begin{array}{c} w^{*}=\sum_{i=1}^{l}\alpha_{i}^{*}y_{i}x_{i},\\ {\;\;b}^{*}=y_{j}-\sum_{i=1}^{l}y_{i}\alpha_{i}^{*}\left(x_{i}\cdot x_{j}\right). \end{array}$$

Consider the following linear regression problem.

The regression hyperplane *y* = (*w* · *x*) + *b* is obtained for the sample set  *T* = {(*x*_1_, *y*_1_),…, (*x*_*l*_^  ^  , *y*_*l*_)}, *x*_*i*_ ∈ *R* ^*n*^, *y*_*i*_ ∈ *R*,  *i* = 1,…, *l*. Intuitively, the difference between the hyperplane value at *x*_*i*_ and the actual input *y*_*i*_ value should be as small as possible. A parameter *ε* > 0 is introduced which requires

(4) min⁡w,b,ε⁡ εs.t. −ε≤yi−((w·xi)+b)≤ε, i=1,…,l.

Equation (4) now needs to be converted into a classification problem. It can be envisaged that all of the sample points in the sample set *T* are, respectively, moved up and moved down *ε* along the *y*_*i*_ direction, so that the sample set *T* ^+^ = {(*x*_*i*_, *y*_*i*_ + *ε*) | *i* = 1,…, *l*} obtained after the upward movement is above the regression hyperplane (positive class) and the sample set *T* ^−^ = {(*x*_*i*_, *y*_*i*_ − *ε*) | *i* = 1,…, *l*} obtained after the downward movement is below the regression hyperplane (negative class point). The regression hyperplane is thus transformed into a partition hyperplane, and the regression problem is converted into a classification problem.

This problem can be solved by modifying (1):

(5) min⁡w,η,b 12||w||2+12η2,s.t. zi((w·xi)+η(yi+ziε)+b)≥1, i=1,…,2l,

where *η* is the weight vector corresponding to the *y* component of the sample points:

(6) zi={1,i=1,…,l;−1,  i=l+1,…,2l.

The partition hyperplane (w* · *x*) + η*y + b* = 0 can be obtained according to the optimal solution (w*, η*, b*) to (5), which corresponds to the regression function *y* = (*w* · *x*) + *b*, where *w* = −w*/η* and *b* = −b*/η*.

Letting ε* = *ε* − 1/η*, (5) is then equivalent to the following:

(7) min⁡w,b 12||w||2,s.t.  (w·xi)+b−yi≤ε∗, i=1,…,l,   yi−(w·xi)−b≤ε∗, i=1,…,l.

The algorithm is further improved by allowing fitting error through the introduction of slack variables *ξ* = {*ξ*_*i*_} and a penalty parameter *C*, which modifies (5) as follows:

(8) min⁡w,η,b,ξ ⁡12||w||2+12η2+Cl∑i=12lξi,s.t.  zi((w·xi)+η(yi+ziε)+b)+ξi≥1,i=1,…,2l,   ξi≥0, i=1,…,2l.

Provided that the optimal solution to (8) is (w*, η*, b*, ξ*), then *ε* = *ε* − 1/η* and (8) is equivalent to

(9) min⁡w,b 12||w||2+Cl∑i=1l(ξi+ξi∗),s.t.  (w·xi)+b−yi≤ε∗+ξi, i=1,…,l,yi−(w·xi)−b≤ε∗+ξi∗, i=1,…,l,ξi,ξi∗≥0, i=1,…,l.

In the objective function of (9), minimizing (1/2)||*w*||^2^ renders the regression function flat and increases the generalizability. (*C*/*l*)∑_*i*=1_^l^(*ξ* + ξ*) is the total error of the regression function and should be minimized.

Setting the solution to (9) as $\left(\bar{w},\bar{b}\right)$, the regression function is then

(10) $$\begin{array}{c} y=\left(\bar{w}\cdot x\right)+\bar{b},\;\bar{w}=-\frac{w^{*}}{\eta^{*}},\;\bar{b}=-\frac{b^{*}}{\eta^{*}}. \end{array}$$

The dual problem is then written as follows:

(11) min⁡α ⁡12∑i,j=1l(αi∗−αi)(αi∗−αi)(xi·xj)  +ε∗∑i=1l(αi∗+αi)−∑i=1lyi(αi∗−αi),s.t. ∑i=1l(αi∗−αi)=0, 0≤αi,αi∗≤Cl,i=1,…,l.

The solution to (11) is derived as $\bar{\alpha_{1}}={\left(\bar{\alpha_{1}},\bar{\alpha_{1}^{*}},\ldots ,\bar{\alpha_{l}},\bar{\alpha_{l}^{*}}\right)}^{T}$. In general, the solutions to (9) and (11) have the following relationship:

(12) $$\begin{array}{c} \bar{w}=\sum_{i=1}^{l}\left(\bar{\alpha_{i}^{*}}-\bar{\alpha_{i}}\right)x_{\text{i}},\\ \bar{b}=y_{j}-\sum_{i=1}^{l}\left(\bar{\alpha_{i}^{*}}-\bar{\alpha_{i}}\right)\left(x_{i}\cdot x_{j}\right)\pm \varepsilon^{*}, \end{array}$$

where *j* is the sample index of the corresponding multiplier $\bar{\alpha }\neq 0\;\;$(when it is positive) or the sample index of corresponding multiplier $\bar{\alpha^{*}}\neq 0$ (when it is negative).

As ε* is a constant that is known in advance in the actual process, simplifying ε* will not have any effect in (9), (11), and (12), and after this replacement the linear SVM machine of the insensitive loss function can be obtained.

For a wide range of nonlinear problems, the SVM maps samples to the high-dimensional Hilbert space by skillfully using nuclear transform methods and processing using linear methods. If the mapping is Φ : *x* → *φ*(*x*), then it can be seen that (11) and (12) are only relevant to the inner product (dot product) of the sample points. Given this characteristic, the actual mapping function Φ need not be known: only the functional relationship of the sample points in the original space in the dot product of the high-dimensional space after mapping needs to be determined, which is represented by the fact that *K*(*x*_*i*_, *x*_*j*_) = (*φ*(*x*_*i*_) · *φ*(*x*_*j*_)).  *K*(*x*_*i*_, *x*_*j*_) is a kernel function. The Gauss radial basis kernel, polynomial kernel, B-spline kernel, Fourier kernel, and Sigmoid kernel are commonly used kernel functions.

The complete algorithm of the SVM for dealing with nonlinear regression problems is as follows.

(1) Select parameters *ε* and *C* and the appropriate kernel *K*(*x*_*i*_, *x*_*j*_).
(2) Construct the following optimization problem:
  (13) min⁡α⁡ 12∑i,j=1l(αi∗−αi)(αj∗−αj)K(xi·xj)  +ε∑i=1l(αi∗+αi)−∑i=1lyi(αi∗−αi),s.t.  ∑i=1l(αi−αi∗)=0,0≤αi,  αi∗≤Cl,i=1,…,l.
 Find the optimal solution $\bar{\alpha }={\left(\bar{\alpha_{1}},\bar{\alpha_{1}^{*}},\ldots ,\bar{\alpha_{l}},\bar{\alpha_{l}^{*}}\right)}^{T}$.
(3) Construct the decision function
  (14) $$\begin{array}{c} f\left(x\right)=\sum_{i=1}^{l}\left(\bar{\alpha_{i}^{*}}-\bar{\alpha_{i}}\right)K\left(x_{i}\cdot x\right)+\bar{b}, \end{array}$$
 where $\bar{b}=y_{i}-\sum_{i=1}^{\text{l}}\left(\bar{\alpha_{i}^{*}}-\bar{\alpha_{i}}\right)\left(x_{i}\cdot x_{j}\right)\pm \varepsilon$; the positive sign is selected when $\bar{\alpha_{j}}\neq 0$, and the negative sign is selected when $\bar{\alpha_{i}^{*}}\neq 0$.

### 2.5. Construction of the SVM Regulation Model 

The SVM method is applied to predict the reclamation effect of a desulfurization byproduct (gypsum) on sodic soils. The main factors influencing the reclamation process must first be determined, and then the sample datasets of observations are selected for training the SVM. Predictions can then be made according to the parameters obtained from the training.

*Determination of the Factors of Influence.* The reclamation of sodic soils with desulfurization byproducts is a multi-ion transportation and transformation process that is accompanied by ion exchange-adsorption and precipitation-dissolution reactions. Under one-dimensional vertical infiltration conditions, the factors that affect the soil solute transport include transport time, transport distance, soil texture, soil bulk density, soil moisture, ion concentration in the soil solution, ion concentration in the supply solution, and ion concentration in the solid exchange phase. When the ion concentration in the soil solution, ion concentration in the solid exchange phase, soil texture, soil bulk density, and soil texture are definite, the ion concentration in the supply solution, transport time, and transport distance can be used as the basis for predicting the soil solute transport. After determining the soil solution concentration in multiple profiles in a one-dimensional vertical transport experiment and training the model in terms of the transport time, transport distance, and corresponding soil solute content, the soil solute transport SVM control model can be established to simulate the spatial and temporal variations of the soil solute transport.

*Construction of the Sample Dataset.* To eliminate the effect of each factor caused by differences in dimension and units, the input and output parameters of the sample are normalized using the following equation:

(15) $$\begin{array}{c} y_{i}=\frac{2\left(z_{i}-z_{\min}\right)}{z_{\max}-z_{\min}}-1, \end{array}$$

where *z*_*i*_ and *y*_*i*_ are the variables before and after normalization, respectively, and *z*_min⁡_ and *z*_max⁡_ are the maximum and minimum values, respectively, of the sample data *z*.

*Learning and Training the SVM.* The Matlab SVM toolbox is used to build and train the model. The main steps in this process include inputting the input and output parameter files, selecting the kernel function and kernel parameter, and data training and testing.

## 3. Results

### 3.1. Ca^2+^ and Na^+^ Concentration in the Leachate

**Figure 2:**
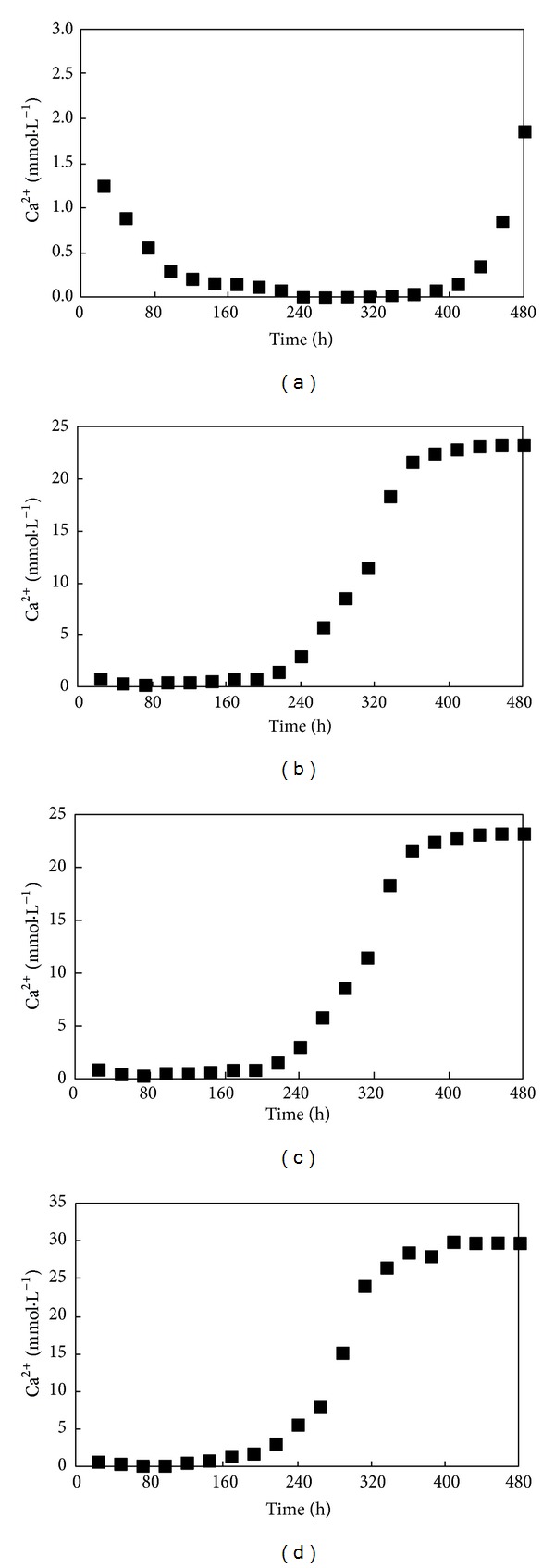
Changes in the measured values of Ca^2+^in the leachate with leaching times ((a)* T*1; (b)* T*2; (c)* T*3; (d)* T*4). The explanation of each condition (*T*1–*T*4) is reported in Table 2.

The measured values for the Ca^2+^ concentrations in the leachate are shown in Figure 2. When the Ca^2+^ concentrations in the supply water were 14.71, 22.06, and 29.41 mmol·L^−1^, Ca^2+^ penetration was realized. When the Ca^2+^ concentration in the supply water was higher, Ca^2+^ penetration was faster. Ca^2+^ did not penetrate when the Ca^2+^ concentration was 7.35 mmol·L^−1^. In the initial stage, because the soil solution itself contained a certain amount of soluble Ca^2+^, the Ca^2+^ in the supply water first reacted with the sodic salts and exchangeable Na^+^ in the soil colloid in the top soil. Before the Ca^2+^ reached the bottom of the soil column, the Ca^2+^ in the soil solution was constantly leached from the soil column and the Ca^2+^ concentration in the leachate gradually decreased. However, as the Ca^2+^ gradually reached the bottom of the soil column, the Ca^2+^ concentration in the leachate gradually increased until it reached the Ca^2+^ concentration in the supply water, at which point it stabilized and realized penetration.

**Figure 3:**
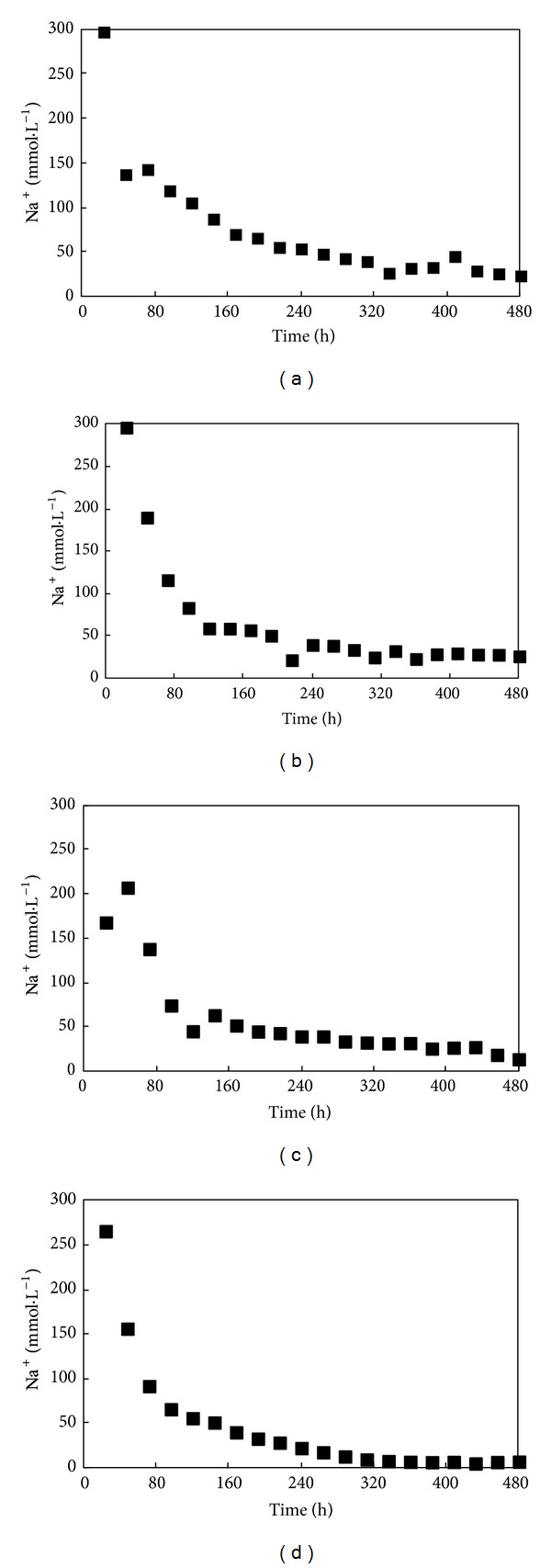
Change in the measured values of Na^+^ in the leachate with leaching time ((a)* T*1; (b)* T*2; (c)* T*3; (d)* T*4). The explanation of each condition (*T*1–*T*4) is reported in Table 2.

The measured values for the Na^+^ concentrations in the leachate are shown in Figure 3. Because there was no Na^+^ in the supply water, the Na^+^ concentration in the leachate gradually decreased. Under higher leachate concentrations, the decrease in Na^+^ was faster due to the increase in the soil's hydraulic conductivity capacity.

### 3.2. Prediction of the Ca^2+^ Concentration in a Penetration Experiment

An SVM model of the variations in Ca^2+^ concentration in sodic soils in a penetration test was constructed and used for data prediction. The training process and analytical results are as follows.

The measured Ca^2+^ concentrations of the soil leachate from 0 h to 480 h in 0.5 g·L^−1^, 1.0 g·L^−1^, and 2.0 g·L^−1^ treatments of gypsum and from 240 h to 480 h in a 1.5 g·L^−1^ treatment of gypsum were used as the input data. The measured Ca^2+^ concentrations of the leachate from 0 h to 240 h in a 1.5 g·L^−1^ gypsum treatment were used as the inspection data. The data were normalized, and the prediction model based on the SVM was constructed in Matlab with the following parameters: kernel function rbf (shaped as in (16)), *δ* = 0.8, *C* = 100, *ε* = 0.001, and

(16) exp⁡(−((x−x′)∗(x−x′))(2δ2)).

**Figure 4:**
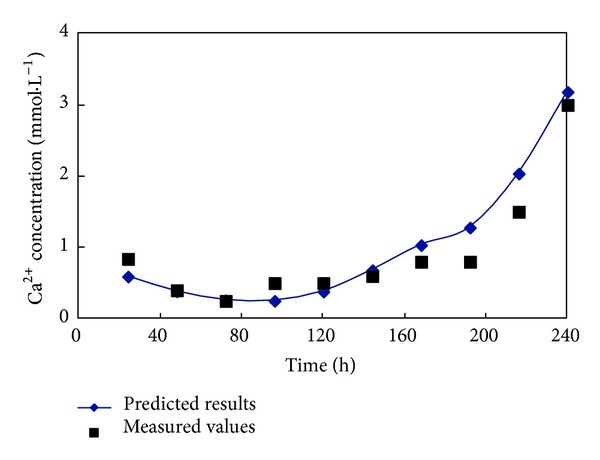
Comparison of the measured values and predicted results for the Ca^2+^ concentration of the soil leachate in a penetration experiment.

**Figure 5:**
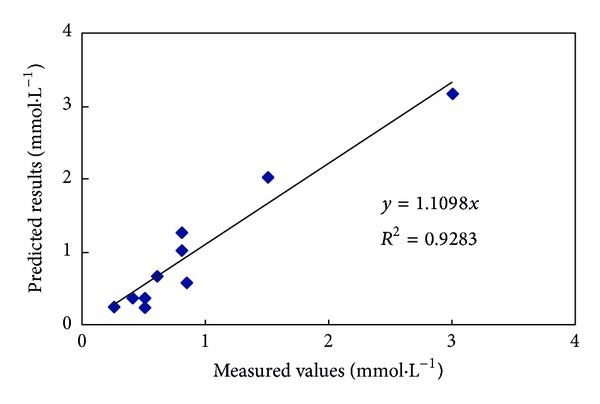
Regression analysis of the measured values and predicted results for the Ca^2+^ concentration of the soil leachate in a penetration experiment.

The SVM model was applied to predict the data. The prediction results and measured data are shown in Figure 4. The results of a regression analysis of the measured data and prediction data are shown in Figure 5. The results show the measured data and prediction data to be strongly correlated. Clearly, the SVM model is able to correctly represent the changes in the Ca^2+^ concentration of the soil leachate in the reclamation process.

### 3.3. Prediction of the Na^+^ Concentration in a Penetration Experiment

The measured Na^+^ concentrations of the soil leachate from 0 h to 480 h in 0.5 g·L^−1^, 1.0 g·L^−1^, and 2.0 g·L^−1^ treatments of gypsum and from 240 h to 480 h in a 1.5 g·L^−1^treatment of gypsum were used as the input data. The measured Na^+^ concentrations of the leachate from 0 h to 240 h in a 1.5 g·L^−1^ gypsum treatment were used as the inspection data. The data were normalized, and the prediction model based on the SVM was constructed in Matlab with the following parameters: kernel function rbf (shaped as in (16)), *δ* = 1.0, *C* = 100, and *ε* = 0.001.

**Figure 6:**
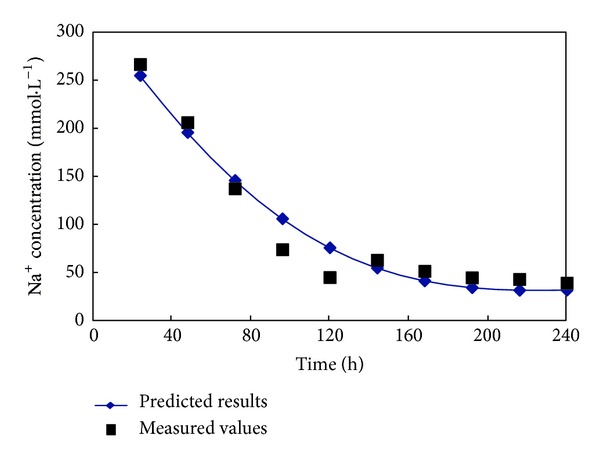
Comparison of the measured values and predicted results for the Na^+^ concentration of the soil leachate in a penetration experiment.

**Figure 7:**
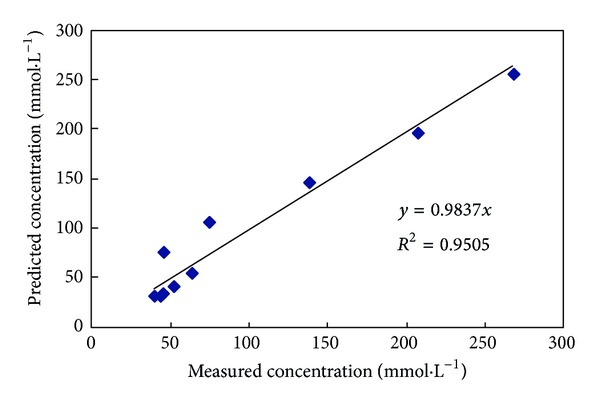
Regression analysis of the measured values and predicted results for the Na^+^ concentration of the soil leachate in a penetration experiment.

The SVM model was applied to predict the data. The prediction results and measured data are shown in Figure 6. The results of a regression analysis of the measured data and prediction data are shown in Figure 7. The results show the measured data and prediction data to be strongly correlated. Clearly, the SVM model is able to correctly represent the changes in the Na^+^ concentration of the soil leachate in the reclamation process.

## 4. Discussion

### 4.1. Validation of the SVM Model

The SVM method is a type of lumped parameter prediction method and has broad applicability in determining small changes in a parameter system [11, 18]. In situations where there is a lack of experimental data and system structure, such models are often able to make acceptable simulations and predictions of ion concentrations [6]. This study introduces the SVM to the complex ion change process that occurs during the application of desulfurization byproducts such as gypsum to improve sodic soils and achieves good prediction and simulation results. In addition to a visual check, measured values of Ca^2+^ and Na^+^ were compared with the results of SVM model prediction using the mean absolute error and the root mean square error. The mean absolute error (MAE) given by [19]

(17) $$\begin{array}{c} \text{MAE}=\frac{1}{n}\sum_{i=1}^{n}\left|{X_{m}}_{i}-{X_{s}}_{i}\right| \end{array}$$

describes the difference between the measured values (*X*_*m*__*i*_) and prediction results (*X*_*s*__*i*_) in the units of a particular variable, with *n* being the number of measurement of Ca^2+^ and Na^+^. The root mean square error (RMSE) given by [19]

(18) $$\begin{array}{c} \text{RMSE}=\sqrt{\frac{\sum_{i=1}^{n}{\left({X_{m}}_{i}-{X_{s}}_{i}\right)}^{2}}{n-1}} \end{array}$$

is the square root of the mean square error, also given in the units of a particular variable. In general, RMSE ≥ MAE. The degree in which the RMSE value exceeds MAE is usually a good indicator of the presence and extent of outliers, or the variance of the differences between the predicted and observed values [19]. The RMSE between the measured values and prediction results of Ca^2+^ in leachate were 0.27 mmol·L^−1^ and 15.91 mmol·L^−1^ for Na^+^. All the RMSE were more than MAE in different degrees, but the degree in which the RMSE value exceeds MAE is low. So, the use of SVM model for prediction of ion transport in sodic soils reclaimed by CaSO_4_ was reasonable.

### 4.2. Comparison with ANN Model

The application presented in the current paper was compared to a very well-known machine learning tool, the artificial neural network (ANN) model. The SVM can take full advantage of the distribution characteristics of the training sample, constructs a discriminant function according to the training sample, and does not need much prior information, which favors it over other nonlinear methods [18, 20]. The SVM finds the best compromise between model complexity and learning ability where sample information is limited and is highly generalizable. The ANN model uses a least squares loss function unlike SVMs which use *ε*-insensitive loss function as a fitness measure [6]. ANN method is more like an art and needs significant skill to obtain a relatively satisfactory result [21, 22]. ANN predictions are not stable and depend on the averages from various network initializations, which may yield a different result each time a model is trained. Converting the SVM algorithm into a quadratic optimization problem should in theory result in a globally optimal solution being obtained [18]. This effectively prevents the local minima problem that neural networks easily fall into, cleverly solves high-dimensional problems by nonlinear transforms and nuclear functions, makes the complexity of the algorithm independent of the sample dimension, and speeds up the training and learning. By contrast, the SVM results are stable and unique.  Table 3 shows the results of the comparison between the SVM and ANN methods based on simulated and predicted data. The RMSE using the SVM method was 0.27 mmol·L^−1^ and 15.91 mmol·L^−1^ for Ca^2+^ and Na^+^, respectively, but those of ANN were 0.52 mmol·L^−1^ and 26.15 mmol·L^−1^, indicating that SVM predicted better results than ANN. The maximum error, minimum error, and average error of the SVM results are smaller than those of the ANN [19]. Therefore, the SVM method has better capability to predict ion transport in sodic soils reclaimed by CaSO_4_.

## 5. Conclusion

The simulation and prediction model of solute transport was constructed using Matlab toolbox based on nonlinear SVM theory, and the transport and transformation law of Ca^2+^ and Na^+^ in Ca^2+^ penetration process was carried out to simulate and predict. The following conclusions can be drawn from our findings.

1. The change of Ca^2+^ and Na^+^ in leachate can be reflected by SVM soil solute transport model by using Matlab toolbox, and the prediction accuracy is high.
2. Using SVM model to predict the spatial and temporal variations of soil solute content is feasible, and it does not require a specific mathematical model. SVM model can take full advantage of the distribution characteristics of training samples and does not require too much of a priori information and use skills.
3. The workload of soil solute transport prediction model based on SVM was greatly reduced without determination of the hydrodynamic dispersion coefficient and retardation coefficient, and the SVM model has a strong practicality.

## Figures and Tables

**Table:** (a)

Exchangeable cations/cmol_c_·kg^−1^					Soil bulk	pH_1:5_
Na^+^	K^+^	Ca^2+^	Mg^2+^	CEC	Density/g·cm^−3^	
8.65	0.60	0.50	1.27	11.02	1.45	9.15

**Table:** (b)

Soluble cations/mmoL_c_·l^−1^				Soluble anions/mmol_c_·l^−1^			
Na^+^	K^+^	Ca^2+^	Mg^2+^	Cl^−^	SO_4_^2−^	HCO_3_^−^	CO_3_^2−^
303.15	5.18	3.33	3.33	193.33	50.00	51.67	20.00

**Table:** (c)

Particle size distribution/%			EC_1:5_
2.0–0.02	0.02–0.002	<0.002	dS·m^−1^
23.2	0.95	42.1	0.95

**Table 2:** Experimental treatments of Ca^2+^ penetration.

Experimental treatments	*T*1	*T*2	*T*3	*T*4
Concentration of CaSO_4_/g·L^−1^	0.5	1.0	1.5	2.0
Concentration of Ca^2+^/mmol_c_·L^−1^	7.35	14.71	22.06	29.41

**Table 3:** Statistical parameters indicative of model performance.

Model	Ca^2+^ concentration		Na^+^ concentration	
	The root mean square error (RMSE)	The mean absolute error (MAE)	The root mean square error (RMSE)	The mean absolute error (MAE)
Support vector machine (SVM)	0.27	0.21	15.91	12.84
Artificial neural network (ANN)	0.52	0.39	26.15	18.81
